# Repeatability and agreement of ultrasonography with computed tomography for evaluating forefoot structure in the coronal plane

**DOI:** 10.1186/s13047-017-0198-1

**Published:** 2017-04-14

**Authors:** Keisuke Matsubara, Tomofumi Matsushita, Yuto Tashiro, Seishiro Tasaka, Takuya Sonoda, Yasuaki Nakayama, Yuki Yokota, Yusuke Suzuki, Mirei Kawagoe, Tomoki Aoyama

**Affiliations:** Department of Physical Therapy, Human Health Sciences, Graduate School of Medicine, Kyoto University, 53 Kawahara-cho Shogoin Sakyo-ku, Kyoto, 6068507 Japan

**Keywords:** Forefoot structure, Ultrasound, Agreement with CT, Repeatability, Coronal plane

## Abstract

**Background:**

Forefoot structure is important to understand some foot problems such as hallux valgus and metatarsalgia. Ultrasonography (US) is a highly portable, noninvasive, low cost, and fast imaging method, especially when compared to magnetic resonance imaging (MRI), computed tomography (CT), and radiography. As the use of US for evaluating forefoot bony structure has not been validated, except for the presence of synovitis, erosions and bursitis within the forefoot in people with inflammatory arthritis, the purpose of this study was to determine whether US is a reliable method for evaluating forefoot structure.

**Methods:**

Sixty feet (30 women, age = 40.1 ± 11.8 years) were examined by US and CT to assess agreement with CT and repeatability of US evaluation of the 2nd metatarsal head height, length between the medial sesamoid bone and 5th metatarsal head, transverse arch height, transverse arch index, sesamoid rotation angle, and area under the transverse arch. The measurement data were evaluated for agreement with CT using the intra-class correlation coefficient (ICC)_3, 1_, Pearson correlation coefficient, and Bland-Altman plot, and with ICC_1, 1_ for repeatability.

**Results:**

The ICC_3, 1_ values of 0.78–0.89, Pearson correlation coefficient of 0.78–0.90, and Bland-Altman plots showed almost perfect agreements between the US and CT method for all parameters, except the area under the transverse arch (AUTA). The ICC_1, 1_ also showed perfect agreements (0.84–0.92) between two sets of US measurements in all parameters.

**Conclusions:**

The US evaluation of forefoot structure in the coronal plane showed good agreement with CT and repeatability of two ultrasonograms in adult women. This reliable evaluation method of forefoot structure can contribute to a quick clinical assessment screening for risk factors of foot problems such as hallux valgus and metatarsalgia. However, because of some limitations such as a lack of inter-observer reliability, more research is needed to validate US evaluation of forefoot structure.

**Trial registration:**

The current study (trial registration number: R0297) was approved by the Ethical Committee for Human Experiments of Kyoto University (http://www.ec.med.kyoto-u.ac.jp) on December 3, 2015. The first participant in this study was enrolled on November 17, 2015 and retrospectively registered.

## Background

The metatarsal and sesamoid bones of the forefoot bear most of the pressure on the plantar surface during gait [1, 2]. Many conditions such as hallux valgus, medial tibial stress syndrome, and diabetes affect the forefoot due to the loading and alignment of these bones [3–6]. Hallux valgus causes sesamoid bone pronation [7], and along with medial tibial stress syndrome [6], it affects transverse arch height [5] as well. In particular, sesamoid bones bear load of up to 300% of body weight [2], and sesamoid position contributes to the distribution of plantar pressure [8, 9], which is associated with foot pain [10]. Metatarsal bones in the coronal plane form the transverse arch, which changes during gait; of these bones, the 2nd metatarsal absorbs the most shock during gait [11, 12]. This pressure on the 2nd and 3rd metatarsal heads (MTH) destabilizes the 2nd metatarsophalangeal joint and is recognized as a cause of metatarsalgia [13]. Therefore, the 2nd MTH height is especially valuable in terms of foot function as a transverse arch and foot disease. Reduced plantar tissue thickness under the MTH has been found in patients with high peak plantar pressures in the high-risk diabetes population [14] and in patients with lesser toe deformities [15]. These foot disorders often decrease quality of life due to pain; however, the mechanism of them are unknown. Evaluation of forefoot structure would provide insight into the mechanism underlying forefoot function and its relationship to foot disorders.

Diagnostic techniques such as magnetic resonance imaging (MRI), computed tomography (CT), and radiography have been used to evaluate forefoot structure. MRI has been used to assess the 1st metatarsophalangeal joint structure, including bone, tendon, and cartilage, and sesamoid bone alignment [16, 17]. CT and radiography have been used to evaluate sesamoid bone heights, metatarsal heads [11, 18], and sesamoid bone rotation angle [7]. CT is especially effective for evaluating bony anatomy because of its high spatial and contrast resolutions for bone [19–21], and it is validated as having high accuracy and precision for foot measurements [22]. However, these are relatively high-cost diagnostic methods, requiring large spaces for equipment and exposing patients to radiation.

Recent technological advances have improved ultrasonography (US) imaging quality, which has enabled musculoskeletal ultrasonography to have diagnostic use [23]. Given its low cost, portability, and real-time diagnostic power, its use has greatly increased. US is a non-invasive and non-ionizing imaging method unlike CT and radiography, which expose patients to radiation. Because of these advantages, US is a more convenient imaging technique, with less burden on patients than other methods. Therefore, it is meaningful that US could be an alternative to other imaging methods. Moreover, by taking advantage of its adaptability, US can be applied to various systems to evaluate forefoot structure with loading [24] and during gait [25].

As for the transverse arch, Kudo et al. [6] and Duerinck et al. [12] evaluated it in terms of the 2nd MTH height and length between the 1st MTH and 5th MTH. In their methods, the transverse arch, which is a bony alignment of MTHs and metatarsals, included the soft tissue since the measurement included the surface of the foot. Hence, the transverse arch should also be evaluated with bony alignment excluding soft tissue to clarify which factor of soft tissue or bony alignment affects the function of the transverse arch. Regarding the sesamoid position, it has been evaluated in the transverse or coronal plane using radiography or CT to study hallux valgus. Kuwano et al. [7] reported that the sesamoid rotation angle in the coronal plane had a higher correlation with the hallux valgus angle than other parameters used to evaluate the sesamoid position. Due to the association between these factors of forefoot plantar structure evaluated in static condition and foot disorder and forefoot function, especially in gait, it is meaningful to evaluate them statically.

Some studies have used US to evaluate plantar soft tissue thickness [26] and forefoot structure [24, 27, 28], such as the 2nd MTH height during gait with the original device which is a platform with an US probe underneath it [25], but its validity is not supported in the literature. The purpose of this study was to establish the agreement of US with CT as a validated method for evaluating forefoot structure. We compared US to CT to determine its reliability for evaluating bony anatomy, and examined the agreement with CT and repeatability of US evaluation of 2nd MTH height, medial sesamoid and 5th MTH (MS-5thMTH) length, transverse arch height (TAH), transverse arch index (TAI), sesamoid rotation angle (SRA), and area under the transverse arch (AUTA) in adult women because of the higher prevalence of foot problems in women [29, 30].

## Methods

### Participants

Thirty women (age = 40.1 ± 11.8 years) with 60 ft were recruited by staff of Kyoto Hakuaikai Hospital to participate in this study, and they received an incentive for volunteering. We included participants in accordance with the inclusion criteria: adult women without a history of foot surgery, congenital disorders, or systemic diseases. Demographic data of participants (Table 1) were investigated by a self-reported questionnaire. The hallux valgus angle, the angle between the first metatarsal and the proximal hallux phalanx, was measured using a goniometer in barefoot standing position. No participants had lesser toe deformities. We obtained written informed consent from each participant after explaining the aim and all study procedures. This study was performed in accordance with the current local guideline and Declaration of Helsinki, and it was approved by the Ethical Committee for Human Experiments (R0297) of Kyoto University.

**Table 1:** Demographic data of participants and feet

Participants (*n* = 30)	
age (year)	40.1 ± 11.8
height (cm)	161.6 ± 19.8
weight (kg)	54.9 ± 8.8
BMI (kg/m^2^)	21.5 ± 4.0
Feet (*n* = 60)	
hallux valgus angle (°)	16.0 ± 8.4
hallux valgus foot	16 (26.7%)

Values are presented as a mean ± standard deviation

Abbreviation: *BMI* body mass index

Hallux valgus foot: the hallux valgus angle is 20° or more

### US measurement

**Fig. 1:**
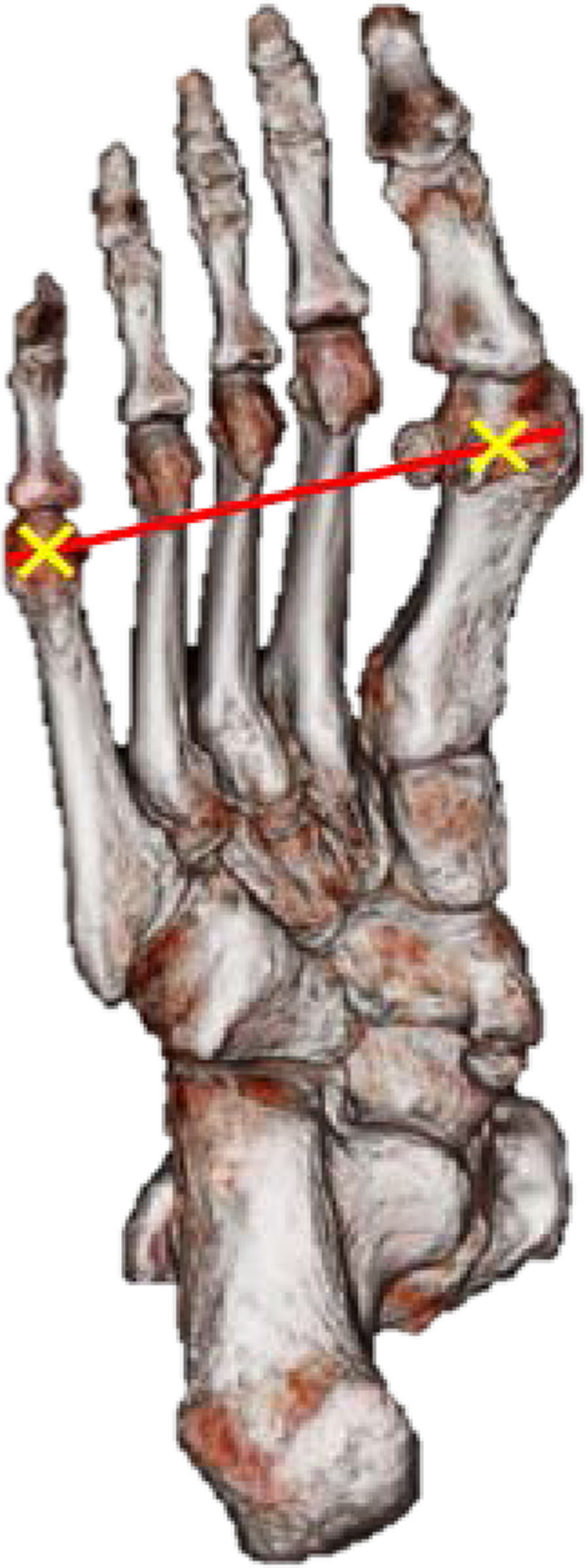
Landmarks for ultrasonography (US) and computed tomography (CT). The 1st metatarsal head (MTH), which includes the sesamoid bones and 5thMTH, were the landmarks for US and CT scans. For US, the probe was located over the line (*red line*) through both landmarks (*yellow marks*). For CT, the image with both landmarks was selected

**Fig. 2:**
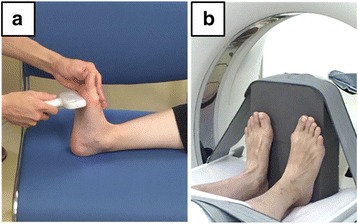
Foot position for ultrasonography (US) and computed tomography (CT). The ankle, proximal interphalangeal joint, and metatarsophalangeal joints are in a neutral position in US and CT scans. **a** The US probe was attached to the landmark line at right angles to the plantar surface. **b** The foot position was fixed using a footboard on the bed of the CT scanner

The examiner (KM) was trained in-house for 3 months under the orthopedist (TA) who had more than 22 years of clinical experience in foot surgery and US evaluation. The US measurement was obtained using a Noblus ultrasound scanner (Noblus, Hitachi Aloka Medical) and a 92-mm wide linear probe at 5–10 MHz (EUP-L53L, Hitachi Aloka Medical) to obtain B-mode images with a maximum view depth of 60 mm, focal depth of 35 mm, gain of 31 dB, and frequency of 7.5 MHz, which were maintained for all US scans. Participants sat upright in chairs with knees extended and ankles in a resting position on footrests without load on the plantar surface. The metatarsophalangeal joints of participants were held in a neutral position, which was the same position as that during CT. The researcher held the participants’ toes only to maintain the position. The researcher palpated the medial sesamoid bone and 5th MTHs on the plantar surface and marked the centre in the medial-lateral and distal-proximal aspects, and then a line was drawn to connect these landmarks as a reference of the scan area (Fig. 1). Echo jelly (GEL-SCAN-KA, Hitachi Aloka Medical) was applied to the skin over the scan region to improve coupling. The probe was placed along the medial-lateral axis over the landmark line in a plane perpendicular to the plantar surface (Fig. 2). The researcher adjusted the probe placement by watching the screen of US machine to obtain images in which all sesamoid bones and 2nd through 5th MTHs were clearly visible. US was conducted twice within a 5-min interval, and two images in the coronal plane for each foot were imported into ImageJ software (National Institute for Health), which has been generally used to measure the length and angle of structures on images in many studies [31, 32].

### CT measurement

Participants lay in a supine position on the CT scanner bed (Aquilion TSX101A, Toshiba) without load on the plantar surface. Straps and a foot board fixed knees at full extension and ankles in resting position (Fig. 2). The field of view was approximately 320 mm, and 1.0-mm thick CT images of all MTHs were obtained in the coronal plane (120 kV × 300 mA, 512 × 512 matrix). A CT slice of each foot was selected from about 60 images with a 60-mm area and a focus on the center of medial sesamoid bones and the 5th MTH in accordance with the landmarks of US measurements, and it was imported into ImageJ software. These procedures for CT were standardized between participants. CT measurements were obtained within 3 weeks from the US measurements.

### Image analyses

**Fig. 3:**
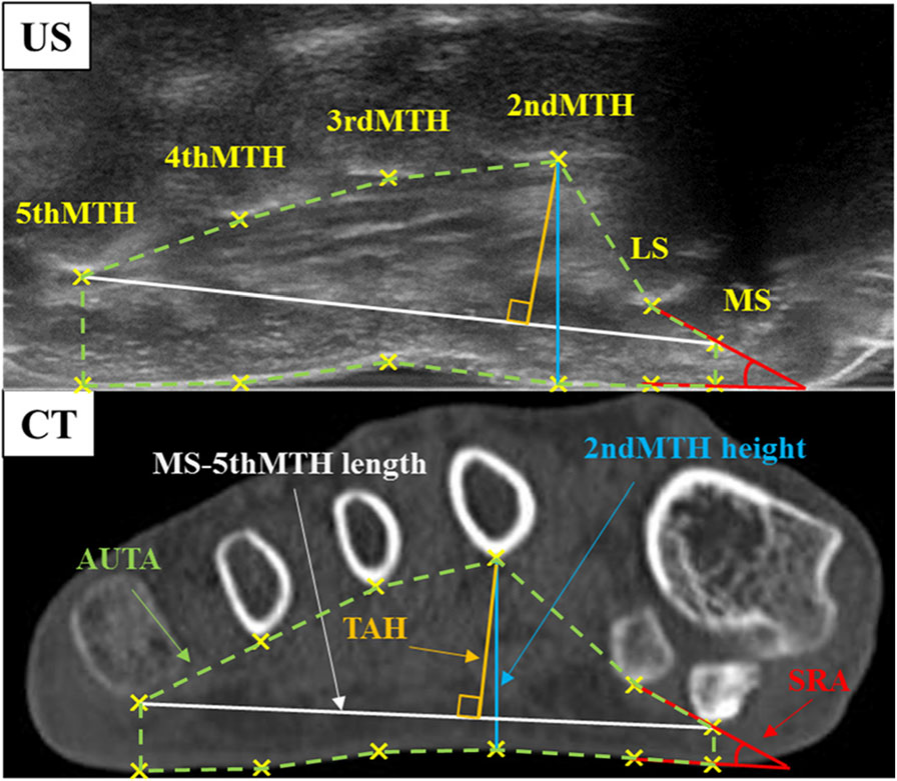
Forefoot structure images in the coronal plane obtained from ultrasonography (US) and computed tomography (CT). The six points of the bones, medial sesamoid bone (MS), lateral sesamoid bone (LS), 2nd metatarsal head (MTH), 3rdMTH, 4thMTH and 5thMTH, were marked. In addition to these six bone points, six plantar points were plotted under each bone point, and all parameters were measured. The length of the *yellow line* indicates the transverse arch height (TAH). The length of the *blue line* indicates the 2ndMTH height. The length of the *white line* indicates the length between the medial sesamoid bone and 5th metatarsal head (MS-5thMTH length). The angle composed of the red lines indicates the sesamoid rotation angle (SRA). The area surrounded by the *green dashed* line indicates the area under the transverse arch (AUTA). As the ultrasound waves cannot penetrate bones, the ultrasonogram of the forefoot demonstrates only the plantar surfaces of sesamoid bones and the 2nd through 5th MTHs

Images obtained by US and CT were transferred to a computer for measurement with ImageJ software. Twelve points were set: six bone points (each lowest point of the epiphysis of the MS, lateral sesamoid bone [LS] and the 2nd through 5th MTHs) and six plantar points at the plantar surface just under each of the six bone points. Six parameters were evaluated: 2ndMTH height (mm), length between the 2ndMTH bone points and plantar distance; MS-5thMTH length (mm), length between MS and 5thMTH points; TAH (mm), length of the perpendicular line through both the MS and the 5thMTH to the 2ndMTH; TAI (%), TAH ÷ MS-5thMTH length × 100; SRA (°), the angle between the line through the two sesamoid bones and the line through the two sesamoid plantar points; AUTA (mm^2^), the area surrounded by the 12 points (Fig. 3). All processes of image analyses, such as the 12-point set and measurement of each parameter, were conducted by one researcher who was blinded to all information regarding the foot characteristics and each image of US and CT.

### Statistical analyses

Agreement of US with the CT method was assessed by evaluating the relationship between CT and the average of two US measurements with the intra-class correlation coefficient (ICC_3,1_), Pearson correlation coefficient, and Bland-Altman plot [33]. In particular, the Pearson correlation coefficient was used to show the proportional relationship between the two methods. For the Bland-Altman plot, differences between the two methods were plotted against their means. Most of the differences were within the limits of agreement (LoA), with a mean difference ± 1.96 standard deviation. The ICC_1, 1_ of two US measurements was evaluated for repeatability. The ICC and Pearson correlation coefficient were calculated using SPSS, version 20.0 software package (IBM Corp.). According to Landis et al., the ICC interpretation scale was classified as follows: below 0.4, poor to fair; 0.41–0.60, moderate; 0.61–0.80, excellent; and 0.81–1, almost perfect [34].

The sample size was calculated for an intra-class correlation coefficient (ICC) of 0.61 to detect at least a significantly moderate level with α error = 0.05 and power = 0.95 using G* power 3.1 software (Heinrich Heine University, Dusseldorf, Germany). As a result, at least 30 samples were needed.

## Results

**Fig. 4:**
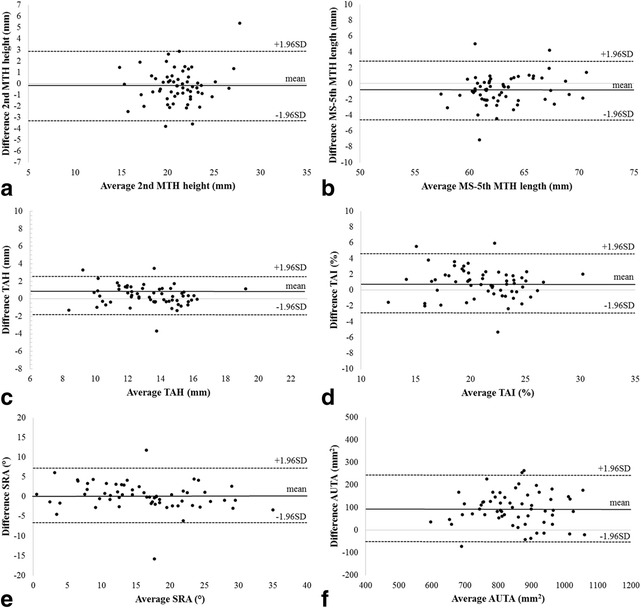
Bland-Altman plots comparing ultrasonography (US) and computed tomography (CT). The solid line shows the mean difference, whreas the dashed line shows 95% limits of agreement (LoA). **a** 2ndMTH height, **b** the length between the medial sesamoid bone and 5th metatarsal head (MS-5thMTH length), **c** transverse arch height (TAH), **d** transverse arch index (TAI), **e** sesamoid rotation angle (SRA) and **f** area under the transverse arch (AUTA)

US and CT measurement results are shown in Table 2. Table 3 shows agreement of US with the CT method, including ICC_3,1_, Pearson correlation coefficient, and the LoA. ICC_3, 1_ values for all parameters, except AUTA, were almost perfect according to Landis et al.’s criteria [30] (2ndMTH height, ICC_3,1_ = 0.83; MS-5thMTH length, ICC_3,1_ = 0.81; TAH, ICC_3,1_ = 0.86; TAI, ICC_3,1_ = 0.84; SRA, ICC_3,1_ = 0.90; AUTA, ICC_3,1_ = 0.78). Mean differences between the two methods (US measurement – CT measurement) were as follows: 2ndMTH height, −0.18 mm; MS-5thMTH length, −0.78 mm; TAH, 0.36 mm; TAI, 0.86%; SRA, 0.19 °; AUTA, 94.82 mm^2^. Bland-Altman plots are shown in Fig. 4; almost all points were within limits of agreement, indicating agreement between US and CT measurements. The ICC_1.1_ of two US measurements were almost perfect in all parameters for repeatability (Table 3).

**Table 2:** US and CT measurement

	US			CT
	Trial 1	Trial 2	Average	
2ndMTH height (mm)	21.2 ± 2.9	20.8 ± 2.8	21.0 ± 2.8	21.2 ± 2.6
MS-5thMTH length (mm)	62.6 ± 3.3	62.7 ± 3.3	62.7 ± 3.2	63.4 ± 2.8
TAH (mm)	13.4 ± 2.1	13.4 ± 2.2	13.4 ± 2.1	13.1 ± 2.2
TAI (%)	21.6 ± 3.5	21.4 ± 3.6	21.5 ± 3.4	20.7 ± 3.6
SRA (°)	16.1 ± 8.1	16.2 ± 7.1	16.1 ± 7.3	15.9 ± 8.1
AUTA (mm^2^)	902.0 ± 120	891.3 ± 112.7	896.7 ± 112.4	801.9 ± 111.4

Values are presented as a mean ± standard deviation

Abbreviations: *MTH* metatarsal head, *MS-5thMTH length* the length between the medial sesamoid bone and 5th metatarsal head, *TAH* transverse arch height, *TAI* transverse arch index, *SRA* sesamoid rotation angle, *AUTA* area under the transverse arch

**Table 3:** Intra-rater agreement of the US measurement and agreement scores between US and CT measurements

						Limits of agreement (95% CI)	
	ICC_1,1_ (95% CI)	ICC_3,1_ (95% CI)	*r*	Mean (95% CI)	Difference (95% CI)	Lower	Upper
2nd MTH height (mm)	0.88 (0.79, 0.92)	0.83 (0.74, 0.90)	0.80	21.13 (20.49, 21.77)	−0.18 (0.39, 1.53)	−3.24 (−3.63,−2.86)	2.88 (3.27, 2.49)
MS-5thMTH length (mm)	0.92 (0.87, 0.95)	0.81 (0.70, 0.89)	0.79	63.05 (62.32, 63.78)	−0.78 (−1.27,−0.32)	−4.55 (−5.02,−4.07)	2.98 (2.48, 3.43)
TAH (mm)	0.84 (0.74, 0.90)	0.86 (0.78, 0.91)	0.86	13.26 (0.52, 2.06)	0.36 (0.64, 0.08)	−1.90 (−1.61,−2.18)	2.62 (2.33, 2.90)
TAI (%)	0.87 (0.80, 0.92)	0.85 (0.78, 0.91)	0.84	21.08 (20.23, 21.94)	0.86 (−2.94, 4.66)	−2.94 (−3.42,−2.46)	4.66 (4.18, 5.14)
SRA (°)	0.85 (0.76, 0.91)	0.89 (0.83, 0.93)	0.90	16.03 (14.15, 17.91)	0.19 (−0.71, 1.09)	−6.90 (−7.80,−6.00)	7.27 (6.38, 8.17)
AUTA (mm^2^)	0.86 (0.78, 0.92)	0.78 (0.66, 0.86)	0.78	849.27 (822.76, 875.78)	94.82 (76.32, 113.32)	−51.39 (−69.89,−32.89)	241.03 (222.53, 259.52)

Abbreviations: *ICC* inter-class correlation coefficient, *CI* confidence interval, *r* Pearson correlation coefficient, *MTH* metatarsal head, *MS-5thMTH length* the length between the medial sesamoid bone and 5th metatarsal head, *TAH* transverse arch height, *TAI* transverse arch index, *SRA* sesamoid rotation angle, *AUTA* area under the transverse arch, *US* ultrasonography, *CT* computed tomography

## Discussion

Regarding the evaluation of forefoot structure, we investigated agreement between US and CT measurements for reliability and intra-rater agreement of two US scans taken at a single time point for repeatability. Based on this investigation, the most important finding of this study was demonstrating agreement with CT and repeatability of forefoot US evaluation (2ndMTH height, MS-5thMTH length, TAH, TAI, SRA, and AUTA) of the feet of adult women without a history of foot surgery, congenital disorders, or systemic diseases. Compared to CT, the LoA indicated good agreement and the ICC_3, 1_ indicated almost perfect correlation, and US showed almost perfect repeatability. Considering that CT evaluation of forefoot structure has been validated [22], US could be estimated to have good validity for evaluating forefoot structure. Some studies have explored the reliability of plantar musculoskeletal evaluations with US for muscles [35], bursitis, erosions, and synovitis [27]; this is the first study known to assess the reliability of US for evaluating bony forefoot structure alignment in detail. These results support the use of US in clinical practice to evaluate forefoot structure in real time, as it is less burdensome to patients than other methods. MRI and CT must be performed in enclosed spaces, which causes burden and stress to the patient, and they are expensive; MRI, especially, takes a long time, and CT emits radiation to patients.

A Bland-Altman plot demonstrated agreement of the US with CT method. Almost all points lay within the LoA, indicating good reliability of US methods for every parameter. Some points fell outside the LoA, likely because some images obtained from the same participant may have reflected different forefoot placement on US and CT. Despite using the same landmarks, spatial differences between the US probe contact angle and the CT scan angle could produce inaccurate measurements. In addition, it is undeniable that the scanned position of metatarsophalangeal joints was the same between US and CT scans, which might also lead to less agreement between US and CT scans because of the difference in the scanned position.

In studies measuring the 2ndMTH height and SRA, Wang et al. [24] and Gooding et al. [28] showed the 2ndMTH height was 13.6 and 14.2 mm, measured by US in an unloaded position; the 2ndMTH height values in our study were larger (21.1 mm by US, 21.3 mm by CT). In our method, the 2ndMTH height was measured at the more proximal part of the 2nd metatarsal because the imaging landmarks in the coronal plane were the 1stMTH and 5thMTH. The more proximal the metatarsal position measured, the higher the value, which produced our relatively higher values. It is also considered that 2ndMTH height might be affected by participants’ characteristics, such as fat volume, muscle volume and foot size, which could be associated with soft tissue thickness. Kuwano et al. [7] compared SRA in patients with hallux valgus to a control group using radiography, and they reported mean SRA values of 29.3° in the hallux valgus group (hallux valgus angle 20° or greater) and 7.4° in the control group. The mean SRA values in our study were 16.0° by US and 15.8° by CT, which were larger than those of the control group in Kuwano et al.’s study. As SRA is greater with hallux valgus [7] and 16 ft (26.7%) with a hallux valgus angle 20° or greater were found in our participants (Table 1), our SRA values were large.

Until now, the transverse arch has been evaluated by measuring only soft tissue. The transverse arch height is affected by plantar muscle and fat pad thickness; however, the TAH indicates transverse arch bony alignment excluding soft tissue. The TAI indicates the transverse arch height adjusted for foot size in the coronal plane, which was defined as the MS-5thMTH length. Length parameters in the foot such as the TAH are affected by foot size; they need to show reliability of the MS-5thMTH length as a foot size in the coronal plane, which is useful to adjust foot structure parameter, and it is a constructional element that can contribute to better reliability of the TAH and TAI. The TAH and TAI are useful for evaluating the transverse arch bony structure and related hallux valgus [5]. The AUTA indicates forefoot bony alignment and soft tissue thickness in the coronal plane for the overall transverse arch. Using the AUTA, it is possible to determine whether the transverse arch is collapsed or the soft tissue under the transverse arch is compacted. The former is when the AUTA has no changes between weight-bearing and non-weight-bearing, and the latter is when the AUTA becomes smaller in weight-bearing than in non-weight-bearing. Therefore, better accuracy of the AUTA measurement would offer a better understanding of the transverse arch function and structural forefoot change in foot deformities due to diabetes, which are associated with plantar soft tissue thickness. Evaluating the transverse arch using these parameters can clarify whether the bony structure or soft tissue affects the function of the transverse arch and foot disease associated with the transverse arch. We therefore propose using TAH, TAI, and AUTA as new parameters for transverse arch evaluation. As it is not yet known how these parameters compare with clinical assessments, these parameters could be tested in future work that assesses dynamic change during gait.

There were some limitations to this study. The interval was short between the two US scans, and the drawn landmark on the plantar surface for US scans was the same for both US scans. The short interval could increase the ICC_1,1_. Although the drawn landmark was only a reference of the initial placement of the US probe, the placement of the US probe and obtainment of the US image was conducted mainly with more attention paid to the screen image of US, regardless of the location of the landmark. Hence, the same landmark within both US scans had less impact on the intra-rater reliability of the US evaluation. There was a certain time gap between the US and CT examinations. However half of the participants were evaluated by both methods within a day, so the time gap was maximally 3 weeks, which might affect agreement between the two methods. This study was limited in that inter-observer reliability of US was not assessed. As US evaluation is dependent on the examiner’s skill, inter-observer reliability may be increased by well-trained examiners. Our research was insufficient to confirm the validity of US evaluation of the forefoot; however, agreement with CT evaluation was confirmed. Considering these limitations, more work needs to be undertaken in the future to determine the validity of US evaluation of the forefoot compared with a clinical assessment.

## Conclusions

This study showed good agreement of US forefoot structure evaluation with CT as a gold standard in adult women. This has value as a non-invasive, convenient, and inexpensive forefoot evaluation in various fields with access to US. US could provide an opportunity to perform a forefoot evaluation that is less burdensome to patients in clinical practice, and it could be useful for foot screening for risk factors such as the SRA and TAH, and 2ndMTH height, which are indicative of hallux valgus and metatarsalgia, respectively. As this study had some limitations such as a lack of inter-observer reliability, short interval for repeatability, and lack of validity, more research should be performed to confirm US evaluation of the forefoot with a high reliability and validity.

## Abbreviations

AUTA: Area under the transverse arch
BMI: Body mass index
CT: Computed tomography
ICC: Intra-class correlation coefficient
LoA: Limits of agreement
LS: Lateral sesamoid bone
MRI: Magnetic resonance imaging
MS: Medial sesamoid bone
MS-5thMTH length: The length between the medial sesamoid bone and 5th metatarsal head
MTH: Metatarsal head
SRA: Sesamoid rotation angle
TAH: Transverse arch height
TAI: Transverse arch index
US: Ultrasound
